# Supplementary material for: Relation extraction between bacteria and biotopes from biomedical texts with attention mechanisms and domain-specific contextual representations
Source: BMC Bioinformatics. 2019 Dec 3;20:627. doi: 10.1186/s12859-019-3217-3 (PMC6889521; doi:10.1186/s12859-019-3217-3)
Supplement: Supplementary file 1 — Additional file 1 Reimplementation details contain hyper-parameters and training setting for reimplemented TurkuNLP and BGRU-Attn models. [file 12859_2019_3217_MOESM1_ESM.pdf]

Reimplementation Details

Since the source codes or the executables for all previous models except VERSE model were not available and there was no comparison of them with the mean F1 score, we needed to reimplement other existing models with the random seeds to compare with our model. We used Pytorch library to reimplement two existing models, TurkuNLP and BGRU-Attn.

To reimplement TurkuNLP model, we used multiple LSTMs for each type of features as described in the original paper. However, instead of constructing the ensemble of 15 deep learning models, we built only the best-performed model based on the validation dataset. The model was trained for 4 epochs using Adam optimizer and every hyper-parameter was set as in the original paper. Our reimplemented model achieved the maximum F1 score of 51.99% compared to 52.10% which was outlined in the original paper.

To reimplement the BGRU-Attn model, we utilized a BGRU based on Additive attention mechanism over the dynamic extended trees with words, POS tags, and relative distances as its input. Since the method to train domain-oriented word embedding model is not provided in the original paper, we instead employed the word embedding model based on biomedical entities and syntactic chunks using hierarchical softmax method, as described in the author's previous paper[1]. Also, since most of the hyper-parameters were also not given in the original paper, we empirically chose the best hyper-parameters using 3-fold cross-validation. The hidden unit number of GRU was 256 and the number of layers was 3. To avoid overfitting when training, we performed early stopping where the performance was evaluated on the development data. Our reimplemented BGRU-Attn model achieved the maximum F1 score of 55.54% compared to 57.42% reported in the original paper.

For further information, we are pleased to provide the source codes of our experiments on a reasonable request, via the corresponding author's email (peerapon.v@chula.ac.th).

1. Jiang Z, Li L, Huang D, Jin L. 2015 Training word embeddings for deep learning in biomedical text mining tasks. In *Proceedings - 2015 IEEE International Conference on Bioinformatics and Biomedicine, BIBM 2015*.
